# Retinoschisin Facilitates the Function of L-Type Voltage-Gated Calcium Channels

**DOI:** 10.3389/fncel.2017.00232

**Published:** 2017-08-08

**Authors:** Liheng Shi, Michael L. Ko, Gladys Y.-P. Ko

**Affiliations:** ^1^Department of Veterinary Integrative Biosciences, College of Veterinary Medicine and Biomedical Sciences, Texas A&M University College Station, TX, United States; ^2^Texas A&M Institute for Neuroscience, Texas A&M University College Station, TX, United States

**Keywords:** retinoschisin, photoreceptor, retina, L-type voltage-gated calcium channel, X-linked retinoschisis

## Abstract

Modulation of ion channels by extracellular proteins plays critical roles in shaping synaptic plasticity. Retinoschisin (RS1) is an extracellular adhesive protein secreted from photoreceptors and bipolar cells, and it plays an important role during retinal development, as well as in maintaining the stability of retinal layers. RS1 is known to form homologous octamers and interact with molecules on the plasma membrane including phosphatidylserine, sodium-potassium exchanger complex, and L-type voltage-gated calcium channels (LTCCs). However, how this physical interaction between RS1 and ion channels might affect the channel gating properties is unclear. In retinal photoreceptors, two major LTCCs are Cav1.3 (α1D) and Cav1.4 (α1F) with distinct biophysical properties, functions and distributions. Cav1.3 is distributed from the inner segment (IS) to the synaptic terminal and is responsible for calcium influx to the photoreceptors and overall calcium homeostasis. Cav1.4 is only expressed at the synaptic terminal and is responsible for neurotransmitter release. Mutations of the gene encoding Cav1.4 cause X-linked incomplete congenital stationary night blindness type 2 (CSNB2), while null mutations of Cav1.3 cause a mild decrease of retinal light responses in mice. Even though RS1 is known to maintain retinal architecture, in this study, we present that RS1 interacts with both Cav1.3 and Cav1.4 and regulates their activations. RS1 was able to co-immunoprecipitate with Cav1.3 and Cav1.4 from porcine retinas, and it increased the LTCC currents and facilitated voltage-dependent activation in HEK cells co-transfected with RS1 and Cav1.3 or Cav1.4, thus providing evidence of a functional interaction between RS1 and LTCCs. The interaction between RS1 and Cav1.3 did not change the calcium-dependent inactivation of Cav1.3. In mice lacking RS1, the expression of Cav1.3 and Cav1.4 in the retina decreased, while in mice with Cav1.4 deletion, the retinal level of RS1 decreased. These results provide important evidence that RS1 is not only an adhesive protein promoting cell-cell adhesion, it is essential for anchoring other membrane proteins including ion channels and enhancing their function in the retina.

## Introduction

Interactions between extracellular proteins and ion channels can modulate channel gating and function. For example, the interaction between integrin and L-type voltage-gated calcium channels (LTCCs) in smooth muscles is required for stretch-induced contraction in blood vessels (Chao et al., 2011), and in mice lacking the extracellular matrix glycoprotein tenascin-C, the LTCC-dependent form of synaptic plasticity in the hippocampus is impaired (Evers et al., 2002). In the retina, LTCCs are the major calcium channels in retinal neurons, and calcium influx through these channels is essential for cellular calcium homeostasis and neurotransmitter release from photoreceptors, bipolar cells, horizontal cells and amacrine cells (Barnes and Kelly, 2002; Morgans et al., 2005). In addition, LTCCs are involved in the regulation of membrane excitability, resonance properties, endocytosis and synaptic plasticity at reciprocal synapses in these retinal neurons (Palmer et al., 2003a,b; Hull and von Gersdorff, 2004; Vigh et al., 2005; Hull et al., 2006a). Thus, LTCCs participate in multiple retina functions. Two major LTCCs in retinal photoreceptors are Cav1.3 (α1D) and Cav1.4 (α1F): Cav1.3 is present from the inner segment (IS) to the synaptic terminal and is responsible for calcium homeostasis (Firth et al., 2001; Xu et al., 2002; Morgans et al., 2005; Ko et al., 2007), while Cav1.4 is only expressed at the synaptic terminal and is critical in forming photoreceptor ribbon synapses during retinal development (Liu et al., 2013) and is responsible for neurotransmitter release (Strom et al., 1998; Morgans, 2001; Barnes and Kelly, 2002; Morgans et al., 2005; Jia et al., 2014). Mutations of the gene encoding Cav1.4 cause X-linked incomplete congenital stationary night blindness type 2 (CSNB2) in patients (Bech-Hansen et al., 1998; Strom et al., 1998; Zito et al., 2003; Michalakis et al., 2014), while the null mutation of Cav1.3 in mice causes a mild decrease of retinal light responses (Busquet et al., 2010).

Retinoschisin (RS1) is an extracellular adhesion protein secreted mainly from photoreceptors and bipolar cells (Reid et al., 1999, 2003; Reid and Farber, 2005), and it tightly binds to the surface of these cells to maintain retinal cellular organization (Sauer et al., 1997; Wu et al., 2005). RS1 contains discoidin domains that allow itself to form homo-octameric complexes (Wu et al., 2005; Wang et al., 2006; Dyka et al., 2008; Bush et al., 2016; Tolun et al., 2016). In addition, RS1 is able to interact with various molecules on the plasma membrane including phosphatidylserine (Kotova et al., 2010), the sodium/potassium-ATPase and sterile alpha and TIR motif-containing protein (Na/K-ATPase-SARM1) complex (Molday et al., 2007), and avian Cav1.3 (Shi et al., 2009). Mutations in the gene encoding RS1 cause X-linked juvenile retinoschisis (XLRS) that features disorganization of retinal cell layers, disruption of synaptic structures and neurotransmission between photoreceptors and bipolar cells, and progressive photoreceptor degeneration (Weber et al., 2002).

While the physical interaction between RS1 and other molecules is known to be calcium-dependent (Vijayasarathy et al., 2007), the functional significance of these interactions is not clear. How RS1 might regulate ion channel gating properties is not known. Previously, we reported a bi-directional relationship between LTCCs and RS1 in the avian retina: while inhibition of LTCCs blocks RS1 secretion (Ko et al., 2008), RS1 sustains the plasma membrane retention of Cav1.3 in cone photoreceptors (Shi et al., 2009). However, because there are differences between mammalian Cav1.3 and Cav1.4 in their channel biophysical characteristics, retinal distributions and functions, it is not clear whether RS1 might have differential interactions and regulations of the channel gating behaviors of Cav1.3 and Cav1.4. In this report, we determined the biophysical properties of Cav1.3 and Cav1.4 in the presence or absence of RS1. We also determined how the deletion of RS1 might affect the retinal expression of both of these LTCCs. Our data uncovered an important functional link between RS1 and LTCCs.

## Materials and Methods

### Animals

Male C57BL/6J mice at 2–3 months old were used in this study. The Cav1.3^−/−^ mice (C57BL/6J background) were originally developed by Dr. Jörg Striessnig (University of Innsbruck, Innrain, Innsbruck, Austria (Platzer et al., 2000). The Cav1.3^+/−^ (heterozygous) breeding pair for generating Cav1.3^−/−^ (homozygous knockout) were obtained from Dr. Amy Lee (University of Iowa, Iowa City, IA, USA). The Cav1.3^−/−^, Cav1.3^+/−^ and Cav1.3^+/+^ wild type (WT) littermates used in this study were produced at Texas A&M University (College Station, TX, USA). All animal experiments were approved by the Institutional Animal Care and Use Committee of Texas A&M University. Mice were housed under temperature and humidity controlled conditions with 12:12 h light-dark cycles.

### Co-Immunoprecipitation

Fresh porcine eyes were obtained from a local meat processing plant (K&C Meat Processing, Navasota, TX, USA). Retinas were collected and homogenized in 1 ml lysis buffer (1% NP-40). Samples were rotated at 4°C for 3 h to solubilize membrane proteins. Samples were then centrifuged at 14,000 *g* for 30 min at 4°C to remove cell debris, and a small portion of the supernatant was taken for protein (loading control, total ERK) analysis. The rest of the supernatant was pre-cleared with Protein A agarose (GBiosciences, Maryland Heights, MO, USA). The beads were removed and 5 μl of anti-RS1 antibody (Santa Cruz Biotechnology, Dallas, TX, USA) was added and incubated for 3 h. A kit (Pierce/Thermo Fisher Scientific, Waltham, MA, USA) was used to remove heavy and light chain interference. No antibody was added to the control. After antibody incubation, 20 μl Protein A agarose were added to each tube and incubated for another 1.5 h. The beads were collected and processed for Western blot analysis of Cav1.3 (antibody from Alomone, Jerusalem, Israel) and Cav1.4 (antibody generated in Amy Lee’s laboratory, University of Iowa, Iowa City, IA, USA). Western blots were visualized by appropriate secondary antibodies (Cell Signaling, Danvers, MA, USA) and electrochemiluminescence kits (Pierce/Thermo Fisher Scientific, Waltham, MA, USA). A commercially available kit (Bio-Rad, Hercules, CA, USA) following the Bradford method was also used to determine total protein content of the samples. All co-immunoprecipitations (co-IPs) were repeated three times.

### Mammalian Two-Hybrid (Luciferase Reporter) Assays

For the mammalian two-hybrid assay, human full length Cav1.4 (Gene ID: 778; obtained from Amy Lee’s laboratory, University of Iowa, Iowa City, IA, USA) and N-terminus (1-1481bp from ATG) of human Cav1.4 were amplified by PCR (Platinum PCR SuperMix High Fidelity, Thermo Fisher Scientific). The primers for the N-terminal were 5′-aaagtcgactgtcggaatctgaaggcgggaaag-3′ (forward) and 5′-gcatctagaggttttcatgatcttgtttaggca-3′ (reverse). The PCR products were purified (Qiaquick, Qiagen, Germantown, MD, USA) and subcloned into the pGEM-T-easy vector (Promega, Madison, WI, USA) to confirm the sequence. The human full length gene encoding RS1 (Gene ID: 6247; 675 bp from ATG to TGA) was also amplified by PCR from the pCDNA-RS1 plasmid (Shi et al., 2009). The RS1 and Cav1.4 N-terminal were inserted into mammalian expression vectors pBind and pACT, respectively (Promega). The transfection reporter assay was carried out by a luciferase assay system (Promega). Briefly, three constructs, pBind or pBind-RS1, pACT or pACT-Cav1.4-N terminal, and pG5Luc (100 ng each) were cotransfected into Cos1 cells (TransIT^®^-COS transfection kit, Mirus, Madison, WI, USA). After cells were harvested, 10 μl of the supernatant was mixed with luciferase substrate, and the relative luciferase activity was determined by luminosity (Perkin-Elmer, Waltham, MA, USA). All luciferase assays were repeated six times.

### Plasmids

pCDNA-RS1 (Human), pCDNA-RS1(R141G), pCDNA-RS1(W92C) were generous gifts from Dr. Dorothy Trump, University of Manchester, Manchester, UK (Shi et al., 2009). pCAGIG-RS1s (WT, R141G, W92C) were constructed by inserting the RS1 encoding fragment from the pCDNA3.1 vector into pCAGIG (EcoRI). The calcium channel α2δ1 subunit (rat) expression vector was a generous gift from Dr. Terrance P. Snutch (University of British Columbia, Vancouver, BC, Canada). The pCDNA-Cav1.3 α1 subunit (mouse) and pCDNA-Cav1.4 α1 subunit (mouse) were generated in Amy Lee’s laboratory (University of Iowa, Iowa City, IA, USA). The pCMV-Sport-β2 subunit (mouse) was purchased from the MGC cDNA clones collection (Dharmacon, GE, Lafayette, CO, USA). The empty plasmid vector phrGFP containing green fluorescent protein (EGFP) was obtained from Agilent Technologies (Santa Clara, CA, USA). The plasmids were amplified in *E. coli* and purified with a kit (Qiagen). All plasmid sequences were confirmed by DNA sequencing (Gene Technologies Lab, Texas A&M University, College Station, TX, USA).

### Cell Culture and Transfection

Human HEK 293 cells (American Type Culture Collection, ATCC, Manassas, VA, USA) were maintained in Dulbecco’s modified Eagle medium (Lonza, Portsmouth, NH, USA) containing 10% fetal bovine serum (Thermo Fisher Scientific, Waltham, MA, USA) and 100 μg/ml penicillin/100 μg/ml streptomycin (Life Technologies, Grand Island, NY, USA), 1mM sodium pyruvate (Life Technologies) and 1× non-essential amino acids (Life Technologies) at 37°C and 5% CO_2_. The cells were plated and cultured on glass coverslips (12 mm diameter) at 70%–80% confluence 24 h before the transfection. Transfections were performed using a lipofectamine 2000 transfection reagent (Life Technologies) according to the manufacturer’s protocol. Up to 500 ng of DNA (100 ng for each plasmid) was transfected into the cells. All cells were co-transfected with EGFP. The culture media were replaced 12 h after transfections. The cells were recorded 48–60 h after transfections.

### Patch-Clamp Electrophysiology

Whole-cell voltage-clamp recordings of LTCC currents were carried out on transfected cells that expressed GFP. The external solution for barium (Ba^2+^) carried LTCC currents contained the following (in mM): Tris 140, BaCl_2_ 10, MgCl_2_ 1 and glucose 5.6, pH 7.4 adjusted with HCl. The external solution for calcium (Ca^2+^) carried LTCC currents contained the following (in mM): Tris 140, CaCl_2_ 10, MgCl_2_ 1 and glucose 5.6, pH 7.4 adjusted with HCl. The pipette solution was (in mM): Cs acetate 135, CsCl 10, MgCl_2_ 2, EGTA 1.1 and HEPES 10, pH 7.4 adjusted with CsOH. The transfected cells were visualized under a fluorescence microscope (IX71, Olympus America, Center Valley, PA, USA). Cells were recorded using a 200 ms step command with holding potential at −65 mV and steps from −80 mV to 60 mV at 10 mV increments. Currents were recorded at room temperature using a patch-clamp amplifier (Model 2400, A-M Systems, Carlsborg, WA, USA). Signals were low pass-filtered at 2 kHz and digitized at 5 kHz with a Digidata 1550A interface and pCLAMP 10.5 software (Axon Instruments/Molecular Devices, Union City, CA, USA). After gigaohm seals were formed, the electrode capacitance was compensated. The membrane capacitance, series resistance and input resistance of the recorded cells were measured by applying a 5 mV (100 ms) depolarizing voltage step from a holding potential of −65 mV. The membrane capacitance reading was used as the value for whole cell capacitance. The current density (pA/pF) was obtained by dividing the current amplitude (pA) by the membrane capacitance (pF). Currents were leak-subtracted after data acquisition. The conductance-membrane potential relationships were analyzed by fitting the Boltzmann equation: G/Gmax = 1/(1 + exp[Vmid − V/Ka]); G: conductance, V: membrane voltage, Vmid: the membrane potential that elicits half of the maximal activation (current), and Ka: the activation slope factor. The protocol used to determine calcium-induced inactivation (CDI) of LTCCs was based on Peterson et al. (1999). The *r*_30_ is the ratio of remaining currents at the end of 30 ms voltage steps (*I*_30_) against the peak maximal current (*I*_max_) and is used to quantify the level of inactivation. The strength of CDI was further quantified by the parameter f, defined as the difference between *r*_30_ values in Ba^2+^ vs. Ca^2+^ taken at −10 mV (Peterson et al., 1999; Mori et al., 2004; Tan et al., 2012). An f value of 0 indicates that there is no CDI, whereas the maximal f value of 1 indicates a complete CDI.

### Immunohistochemistry

The frozen tissue blocks for sectioning containing Cav1.4 null and WT littermate mouse eyes that were fixed and cryo-protected (as described previously; Liu et al., 2013) were obtained from Amy Lee’s laboratory and sectioned at 12 μm thickness. The frozen RS1 null and control WT mouse retina sections (10 μm) were provided by Dr. Paul Sieving’s laboratory (National Eye Institute, Bethesda, MD, USA), and the processing of the retinal sections was described previously (Takada et al., 2004). The eyes excised from the Cav1.3^−/−^ and WT littermates were fixed in Zamboni fixative (American Matertech Scientific Inc, Lodi, CA, USA) and then cryo-protected in a 30% sucrose-phosphate-buffered saline (PBS) solution. Cav1.3^−/−^ and WT eyes were embedded side by side in Tissue-Tek O.C.T. Compound (Sakura Finetek Inc, Torrance, CA, USA) and stored at −80°C. The frozen eye sections (10 μm) were cut using a cryostat (Leica Biosystem, Buffalo Grove, IL, USA) and mounted on glass slides. After washes with 0.1 M sodium PBS (pH 7.4), the sections were incubated with a blocking solution containing 10% fetal bovine serum in PBS for 2 h at room temperature then incubated with the primary antibody at 4°C overnight. The next day, sections were washed three times with PBS containing 0.1% Triton 100 (PBST), incubated with a secondary antibody at room temperature for 2 h in a dark chamber, then washed with PBST, and mounted with ProLong Gold antifade reagents with 4′,6-diamidino-2-phenylindole (DAPI; Life Technologies). The primary antibodies used were rabbit anti-Cav1.3 (1:100; Alomone), mouse anti-Ribeye (1:100; EMD Millipore, Billerica, MA, USA), rabbit anti-Cav1.4 (1:1000; generated in Amy Lee’s laboratory) and rabbit anti-RS1 (1:100; Santa Cruz Biotechnology, Dallas, TX, USA). The secondary antibodies used were Alexa fluor 488 goat anti-rabbit IgG (1:200; Life Technologies), Cy5 goat anti-mouse IgG (1:200; Abcam, Cambridge, MA, USA) and Texas red donkey anti-goat IgG (1:200; Life Technologies). The images were captured with a Zeiss LSM 780 NLO Multiphoton Microscope (Carl Zeiss AG, Oberkochen, Germany), and the conditions (magnification and exposure time) of images taken from WT or mutant retinal sections for each antibody were identical.

### Statistics

All data are presented as mean ± standard error of the mean (SEM). Student’s *t*-test and one-way ANOVA followed by Tukey’s *post hoc* test for unbalanced n were used for statistical analyses. Throughout, *p* < 0.05 was regarded as significant.

## Results

### There Was a Physical Interaction between RS1 and LTCCα1 Subunits, Cav1.3 and Cav1.4, in the Mammalian Retina

**Figure 1:**
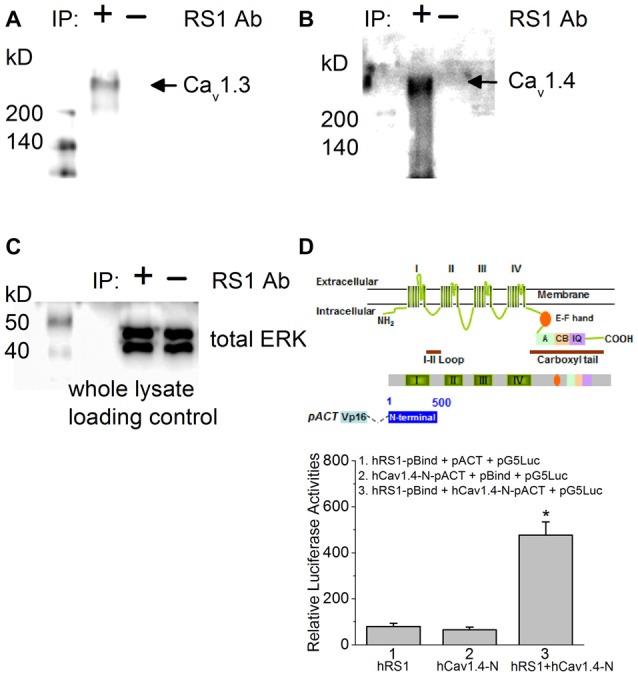
There is a physical interaction between retinoschisin (RS1) and L-type voltage-gated calcium channel (LTCC)α1 subunits. **(A)** Anti-RS1 antibody (RS1 Ab) is able to co-immunoprecipitate Cav1.3 from the porcine retina. **(B)** RS1 Ab is able to co-immunoprecipitate Cav1.4 from the porcine retina. **(C)** The whole cell lysates as loading control for **(A,B)**. **(D)** Mammalian two-hybrid (luciferase reporter) assays show that hRS1 is able to interact with the first 500 amino acids from the N-terminus of Cav1.4 (hCav1.4-N) including the first motif (I). Cells co-transfected with hRS1 and hCav1.4-N (hRS1 + hCav1.4-N) have significantly higher luciferase activities than the other two control groups (*n* = 6 for each group, *p < 0.05, one-way ANOVA with Tukey’s *post hoc* tests. hRS1 vs. hRS1 + hCav1.4-N, *p* = 0.00000199; hCav1.4-N vs. hRS1 + hCav1.4-N, *p* = 0.00000126).

Using porcine retinas, we took advantage of tissue abundance and determined the physical interaction between RS1 and Cav1.3 or Cav1.4 by co-IP. An antibody against RS1 (RS1 Ab) was able to pull down Cav1.3, as well as Cav1.4 (Figures 1A,B), thus providing the first evidence that RS1 interacts with mammalian Cav1.3 and Cav1.4. We next used mammalian two-hybrid (luciferase reporter) assays to determine the interaction between RS1 and Cav1.4. We previously showed that RS1 interacts with the first 500 amino acids from the N-terminal of chicken Cav1.3 containing the first (I) of four (I–IV) homologous motifs that is highly conserved with the human Cav1.3 (Shi et al., 2009). Using a similar strategy to determine the interaction between RS1 and Cav1.4, a full-length human *rs1* cDNA (hRS1) was inserted into a pBIND vector that encoded a recombinant protein with GAL4 DNA binding domain as the bait. The human Cav1.4-N terminal fragment (hCav1.4-N; 500 amino acids) included the short N-terminus, the first motif (I), and a partial junction sequence between the first (I) and second (II) motifs. The hCav1.4-N was inserted into a pACT vector that encoded a protein containing a VP16 activation domain. The pG5Luc contained five tandem GAL4 binding sequences upstream of a luciferase coding region that was used to report protein interactions. The relative luciferase activity was at least four times higher in cells co-transfected with hRS1 and hCav1.4-N compared to the controls (Figure 1D), which supported our co-IP data. Thus, RS1 was able to interact with the first motif of Cav1.4 (Figure 1), as well as Cav1.3 shown previously (Shi et al., 2009). We next investigated the functional interaction between RS1 and Cav1.3 or Cav1.4.

### RS1 Facilitated the Voltage-Dependent Activation of Cav1.3-LTCCs

**Figure 2:**
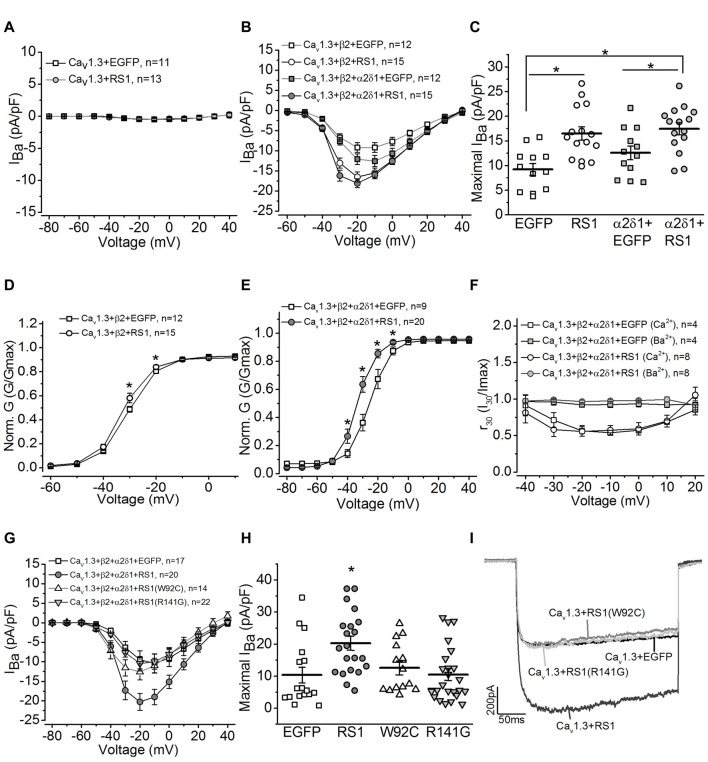
RS1 augments the current density of Cav1.3-LTCC in HEK cells. **(A)** Cells transfected with Cav1.3 subunit without other LTCC auxiliary subunits (Cav1.3 + EGFP) do not have functional LTCCs. Co-transfection with Cav1.3 and RS1 do not elicit LTCC currents carried by Ba^2+^ (*I*_Ba_). **(B)** Cells transfected with Cav1.3 and β2 (Cav1.3 + β2 + EGFP), or Cav1.3, β2, and α2δ1 (Cav1.3 + β2 + α2δ1 + EGFP) display functional Cav1.3-LTCC currents. RS1 significantly enhances Cav1.3-LTCC when co-transfected with functional Cav1.3-LTCC (Cav1.3 + β2 + RS1, or Cav1.3 + β2 + α2δ1 + RS1). **(C)** The maximal current densities (*I*_Ba_) elicited at −20 mV are (in pA/pF): −9.22 ± 1.20 for EGFP (Cav1.3 + β2 + EGFP), −16.51 ± 1.27 for RS1 (Cav1.3 + β2 + RS1), −12.58 ± 1.40 for α2δ1 + EGFP (Cav1.3 + β2 + α2δ1 + EGFP), and −18.06 ± 1.13 for α2δ1 + RS1 (Cav1.3 + β2 + α2δ1 + RS1). *Indicates a significant difference between the groups (*p < 0.05, one-way ANOVA with Tukey’s *post hoc* tests). EGFP vs. RS1, *p* = 0.00138; α2δ1 + EGFP vs. α2δ1 + RS1, *p* = 0.0273; EGFP vs. α2δ1 + RS1, *p* = 0.000260. **(D,E)** Co-transfection with RS1 significantly enhances the voltage-dependent activation of Cav1.3-LTCCs in the presence or absence of α2δ1 subunit. *Indicates that the Cav1.3-LTCC voltage-dependent activation recorded from cells transfected with Cav1.3 + β2 + α2δ1 + RS1 and Cav1.3 + β2 + RS1 are significantly larger than the other two groups without RS1 (*p < 0.05; unpaired Student’s *t*-test). The normalized conductance (Norm. G) is the ratio of conductance (G) against the maximal conductance (Gmax), G/Gmax, and plotted against the elicited membrane voltage (mV). *Indicates a statistical difference between the two groups (*p < 0.05; unpaired Student’s *t*-test). Cav1.3 + β2 + EGFP vs. Cav1.3 + β2 + RS1: *p* = 0.04505 at −30 mV, *p* = 0.02138 at −20 mV. Cav1.3 + β2 + α2δ1 + EGFP vs. Cav1.3 + β2 + α2δ1 + RS1: *p* = 0.0456 at −40 mV, *p* = 0.00278 at −30 mV, *p* = 0.0035 at −20 mV, *p* = 0.01509 at −10 mV. **(F)** RS1 does not alter the calcium-induced inactivation (CDI) of Cav1.3-LTCCs. Cells with functional Cav1.3-LTCC (Cav1.3 + β2 + α2δ1) in the presence or absence of RS1 co-expression were recorded with Ba^2+^ or Ca^2+^ as the Cav1.3-LTCC current carriers. The Cav1.3-LTCC currents carried by Ca^2 +^, but not Ba^2 +^, display CDI. **(G)** Co-transfection with the RS1 mutants (W92C or R141G) and a fully functional Cav1.3-LTCC (Cav1.3 + β2 + α2δ1) does not enhance Cav1.3-LTCC currents. **(H)** The maximal Cav1.3-LTCC current densities (*I*_Ba_) elicited at −20 or −10 mV are (in pA/pF): −10.39 ± 3.44 for EGFP (Cav1.3 + β2 + α2δ1 + EGFP), −20.27 ± 2.19 for RS1 (Cav1.3 + β2 + α2δ1 + RS1), −12.59 ± 2.06 for W92C (Cav1.3 + β2 + α2δ1 + W92C), and −10.47 ± 1.86 for R141G (Cav1.3 + β2 + α2δ1 + R141G). *Indicates that co-transfection with RS1 significantly increases the maximal Cav1.3-LTCC current density compared to the other three groups (*p < 0.05, one-way ANOVA with Tukey’s *post hoc* tests). EGFP vs. RS1, *p* = 0.00893; R141G vs. RS1, *p* = 0.00791; W92C vs. RS1, *p* = 0.052; W92C vs. EGFP, *p* = 0.9655; R141G vs. EGFP, *p* = 0.9981. **(I)** Representative current traces recorded from cells transfected with a fully functional Cav1.3-LTCC (Cav1.3 + EGFP) and co-transfected with Cav1.3-LTCC and RS1 or RS1 mutants (W92C or R141G) are presented.

To determine whether RS1 was able to regulate the Cav1.3-LTCC channel gating behavior, we co-transfected HEK-293T cells with both, and systematically examined how RS1 might modulate Cav1.3-LTCC gating behaviors in the absence or presence of its auxiliary subunits. We first investigated whether RS1 alone was able to elicit calcium influx through Cav1.3 without the auxiliary (β2 and α2δ1) subunits. In the absence of β2 subunit, Cav1.3-mediated currents were not measurable (Figure 2A, Cav1.3 + EGFP), and RS1 was not able to stimulate calcium influx via Cav1.3 (Figure 2A, Cav1.3 + RS1). To further decipher how RS1 might interact with Cav1.3 in the presence of various auxiliary subunits, cells were co-transfected with Cav1.3 and β2 (Cav1.3 + β2) or all auxiliary subunits (Ca1.3 + β2 + α2δ1) concurrently with or without RS1. While α2δ1 does not have any effect on the Cav1 voltage-dependence, this auxiliary subunit enhances the expression of Cav1 + β subunits (Shistik et al., 1995; Bangalore et al., 1996). As expected, α2δ1 mildly increased Cav 1.3-LTCC currents in the presence of the β2 subunit (Ca1.3 + β2 + α2δ1) compared to Cav1.3-LTCCs without α2δ1 (Ca1.3 + β2; Figures 2B,C). We found that co-transfection with RS1 significantly enhanced Cav1.3-LTCC currents in the presence of β2 (Cav1.3 + β2 + RS1; Figures 2B,C), but α2δ1 did not further increase Cav1.3-LTCC currents when RS1 was present (Ca1.3 + β2 + α2δ1 + RS1; Figures 2B,C). Hence, RS1 was able to enhance the Cav1.3-LTCC channel activation that required the presence of β2. The interaction between RS1 and Cav1.3-LTCCs was mainly limited on the α1 subunit (Cav1.3) without interacting with the other extracellular auxiliary subunits (α2δ1).

We next examined whether RS1 was able to enhance the voltage-dependent activation of LTCCs. Co-transfection with RS1 and functional Cav1.3-LTCCs (Cav1.3 + β2 + α2δ1 + RS1 or Cav1.3 + β2 + RS1) significantly facilitated the voltage-dependent activation compared to the Cav1.3-LTCCs without RS1 (Figures 2D,E). RS1 shifted the current-membrane voltage relationship of Cav1.3-LTCCs by −10 mV (Figures 2D,E). One biophysical characteristics of Cav1.3-LTCC is its Ca^2 +^-dependent inactivation (CDI; Peterson et al., 1999; Mori et al., 2004; Tan et al., 2012). When the Cav1.3-LTCC currents are carried by Ca^2 +^, but not Ba^2 +^, Cav1.3-LTCCs display CDI (Figure 2F) with the “f” parameter calculated to quantify the strength of CDI (Peterson et al., 1999; Mori et al., 2004; Tan et al., 2012). We found that RS1 did not affect the CDI of Cav1.3-LTCCs in cells co-transfected with RS1, since there was no statistical difference of the f values between the Cav1.3-LTCCs with or without RS1 (Cav1.3 + β2 + α2δ1: 0.385 ± 0.076; Cav1.3 + β2 + α2δ1 + RS1: 0.402 ± 0.084). Since the CDI of Cav1.3-LTCCs depends on the binding of calcium-calmodulin at the C-terminal of the Cav1.3 (Peterson et al., 1999), these data indicate that RS1 did not interfere with the calcium-dependent structural changes. Through interacting with the first motif, RS1 was able to facilitate the voltage-dependent activation and augment the channel conductance of Cav1.3-LTCCs.

Both W92C and R141G are missense mutations of RS1 detected in XLRS patients (Wang et al., 2002, 2006). The W92C mutation causes cysteine-triggered intermolecular bonding that result in intracellular retention of the mutant RS1 in the endoplasmic reticulum (ER; Wang et al., 2006). The R141G mutation does not interfere with secretion but affects a surface residue within the loop region causing RS1 to lose its ability to bind other molecules (Wang et al., 2006). Neither W92C nor R141G had the ability to augment Cav1.3-LTCCs compared to the WT RS1 (Figures 2G–I). These observations provide evidence that RS1 interacts with Cav1.3-LTCCs and facilitates membrane retention of Cav1.3-LTCCs (Shi et al., 2009), and it further augments the channel voltage-dependent activation without interfering with its calcium-dependent inactivation.

### RS1 Augmented the Voltage-Dependent Activation of Cav1.4-LTCCs

**Figure 3:**
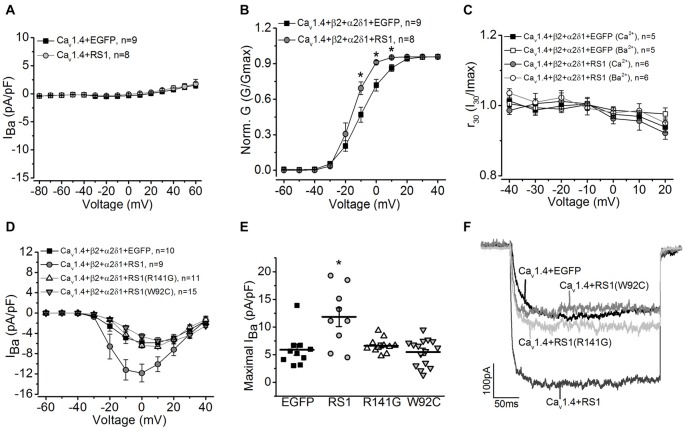
RS1 augments Cav1.4-LTCC in HEK cells. **(A)** Cells transfected with Cav1.4 subunit without other LTCC auxiliary subunits (Cav1.4 + EGFP) do not have functional LTCCs. Co-transfection with Cav1.4 and RS1 do not elicit measurable LTCC currents carried by Ba^2+^ (*I*_Ba_). **(B)** RS1 enhances the channel voltage-dependent activation (Norm. G) of functional Cav1.4-LTCCs. *Indicates a statistical difference between the two groups (*p < 0.05, unpaired Student’s *t*-test). Cav1.4 + β2 + α2δ1 + EGFP vs. Cav1.4 + β2 + α2δ1 + RS1: *p* = 0.02127 at −10 mV, *p* = 0.00322 at 0 mV, *p* = 0.01213 at 10 mV. **(C)** RS1 does not alter the CDI of Cav1.4-LTCCs. Cav1.4-LTCCs are less sensitive to CDI. Cells were recorded with Ba^2+^ or Ca^2+^ as the Cav1.4-LTCC current carrier. **(D)** Cells transfected with Cav1.4 and LTCC auxiliary subunits (β2 and α2δ1) display functional Cav1.4-LTCC currents (Cav1.4 + β2 + α2δ1 + EGFP). Cells co-transfected with RS1 and Cav1.4-LTCC (Cav1.4 + β2 + α2δ1 + RS1) have significantly enhanced Cav1.4-LTCC current density. However, co-transfection with RS1 mutants (either W92C or R141G) and a fully functional Cav1.4-LTCC (Cav1.4 + β2 + α2δ1) does not enhance Cav1.4-LTCC currents. **(E)** The maximal Cav1.4-LTCC current densities (pA/pF) elicited at 0 or 10 mV are: −5.88 ± 0.98 for EGFP (Cav1.4 + β2 + α2δ1 + EGFP), −11.84 ± 1.76 for RS1 (Cav1.4 + β2 + α2δ1 + RS1), −6.61 ± 0.40 for R141G (Cav1.4 + β2 + α2δ1 + R141G), −5.47 ± 0.63 for W92C (Cav1.4 + β2 + α2δ1 + W92C). *Indicates that co-transfection with RS1 significantly increased the maximal Cav1.4-LTCC current density value compared to the other three groups (*p < 0.05, one-way ANOVA with Tukey’s *post hoc* tests). RS1 vs. EGFP, *p* = 0.00105; RS1 vs. R141G, *p* = 0.00363; RS1 vs. W92C, *p* = 0.00013. **(F)** Representative traces from the four groups are shown.

Since the RS1 antibody was able to co-immunoprecipitate Cav1.4 from porcine retinas, and RS1 interacted with the first 500 amino acids of the Cav1.4 N-terminal sequence containing the first motif (Figure 1), we next examined the functional interaction between RS1 and Cav1.4. We applied a similar strategy to systematically examine the interactions between RS1, Cav1.4, and the auxiliary subunits of Cav1.4. Co-transfection with RS1 and Cav1.4 without the β2 subunit did not elicit measurable LTCC currents (Figure 3A). When co-transfected with the full length Cav1.4 and the axillary subunits (Cav1.4 + β2 + α2δ1), RS1 significantly enhanced the Cav1.4-LTCC current density (Figure 3) and the voltage-dependent activation (Figure 3B). Unlike Cav1.2- or Cav1.3-LTCCs, full length Cav1.4 without a deletion of exon 47 shows no discernable CDI (Baumann et al., 2004) and displays unusually slow voltage-dependent inactivation (McRory et al., 2004; Haeseleer et al., 2016). Co-transfection with RS1 and functional Cav1.4-LTCC did not alter the CDI property of Cav1.4-LTCCs (Figure 3C). Likewise, R141G or W92C, missense mutations of RS1, had no impact on functional Cav1.4-LTCCs (Figures 3D–F). Hence, RS1 was able to interact with Cav1.4-LTCCs, enhance its voltage-dependent activation, and augment the channel currents similar to its interaction with Cav1.3.

### Deletion of RS1 Decreased Cav1-LTCCs in the Mouse Retina

**Figure 4:**
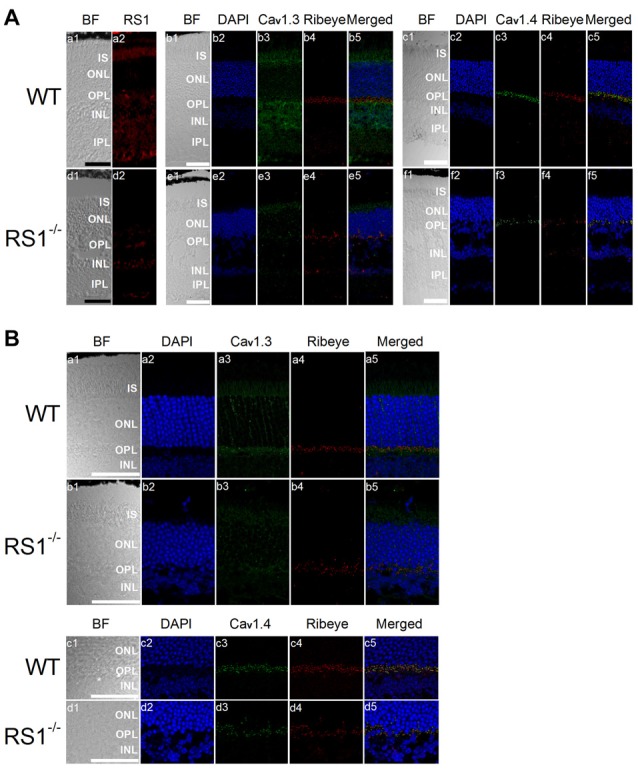
Deletion of RS1 decreases the protein expression of Cav1.3 and Cav1.4. **(A)** The upper panel (a1-c5) contains retinal sections of wild type (WT), and the lower panel (d1-f5) contains retinal sections of RS1^−/−^. (a1-a2) and (d1-d2) are the immunostaining for RS1. (b1-b5) and (e1-e5) are the double immunostaining for Cav1.3 and Ribeye; (c1-c5) and (f1-f5) are the double immunostaining for Cav1.4 and Ribeye. The scale bar represents 50 μm. **(B)** The same immunostained retinal sections are shown at a higher magnification (40×). The upper panel contains retinal sections from WT (a1-a5) and RS1^−/−^ (b1-b5) that were double-stained for Cav1.3 and Ribeye. Images in (a1-a5) and (b1-b5) include retinal layers of IS, ONL, OPL and INL. The lower panel contains retinal sections from WT (c1-c5) and RS1^−/−^ (d1-d5) that were double-stained for Cav1.4 and Ribeye. Images in (c1-c5) and (d1-d5) include retinal layers of ONL, OPL and INL. The scale bar represents 50 μm. 4′*s*,6-diamidino-2-phenylindole (DAPI) stains the nuclei. BF, bright field; IS, photoreceptor inner segments; ONL, outer nuclear layer; OPL, outer plexiform layer; INL, inner nuclear layer; IPL, inner plexiform layer.

Mice lacking RS1 have degenerated retinas that resemble the human XLRS phenotype, which includes schisis cavities in the inner nuclear layer (INL), disorganized outer nuclear layer (ONL) and progressive photoreceptor degeneration (Weber et al., 2002). Immunostaining with an antibody against Ribeye, a marker for ribbon synapses (Schmitz et al., 2000), showed that the outer plexiform layer (OPL) was disorganized in the RS1^−/−^ retina (Figure 4A) indicating that RS1 deletion disrupted synaptic structure. The expressions of Cav1.3 and Cav1.4 were markedly decreased in the RS1^−/−^ retina (Figure 4A). Focusing on changes in photoreceptors, Cav1.3 is normally present in the IS, as well as the ONL and OPL (Figure 4B). In RS1^−/−^ retinas, Cav1.3 was decreased in all three areas (Figure 4B), which verified that degeneration of photoreceptors had taken place. Cav1.4 is mostly located in the photoreceptor synaptic terminals and is responsible for sustained neurotransmitter release from photoreceptors (Morgans, 2001; Liu et al., 2013; Lee et al., 2015). While Cav1.4 existed mainly at the OPL in the WT mouse retina (Figure 4B), it was decreased in the OPL of the RS1^−/−^ retina (Figure 4B). Therefore, deletion of RS1 caused losses of LTCCs (both Cav1.3 and Cav1.4), which could be the consequence of photoreceptor degeneration.

### Deletion of Cav1.4 Decreased RS1 in Mouse Retinas

**Figure 5:**
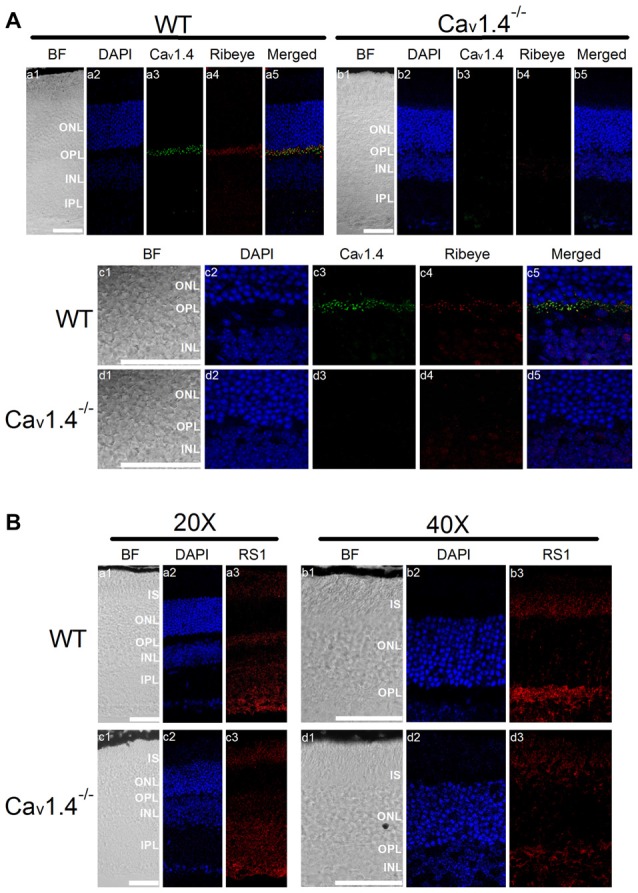
Deletion of Cav1.4 decreases RS1 distribution in the retina. **(A)** The upper panel shows images taken at a lower magnification (20×) of WT (a1-a5) and Cav1.4^−/−^ (b1-b5) retinal sections that were double immunostained with antibodies against Cav1.4 and Ribeye. The lower panel shows images taken at a higher magnification (40×) of WT (c1-c5) and Cav1.4^−/−^ (d1-d5) retinal sections stained for Cav1.4 and Ribeye. The scale bar represents 50 μm. **(B)** The upper panel shows images of WT retina stained with RS1 taken at lower (20×; a1-a3) and higher (40×; b1-b3) magnifications. Likewise, the lower panel shows images of Cav1.4^−/−^ retinal sections stained with RS1 at lower (20×; c1-c3) and higher (40×; d1-d3) magnifications. The scale bar represents 50 μm. DAPI stains the nuclei. BF, bright field; IS, photoreceptor inner segments; ONL, outer nuclear layer; OPL, outer plexiform layer; INL, inner nuclear layer; IPL, inner plexiform layer.

Patients with mutations in the gene encoding Cav1.4 have incomplete CSNB2 (Bech-Hansen et al., 1998; Strom et al., 1998; Zito et al., 2003; Michalakis et al., 2014). Since the retinal light responses recorded by electroretinogram (ERG) of patients with CSNB2 are similar to that of patients with XLRS (Bradshaw et al., 2004), and there was a physical interaction between RS1 and Cav1.4 (Figure 1), we next examined the distribution of RS1 in Cav1.4 null mutant (Cav1.4^−/−^) mouse retinas. No Cav1.4 was detected in the OPL of Cav1.4^−/−^ mouse retinas (Figure 5A), and Ribeye was decreased in the OPL of Cav1.4^−/−^ compared to the WT (Figure 5A). These data confirmed a previous report that mice lacking Cav1.4 have defects in the development of photoreceptor ribbon synapses (Liu et al., 2013; Zabouri and Haverkamp, 2013). While RS1 is normally present in the IS, ONL, OPL and INL (Figure 5B), its expression was reduced in all retinal layers in the Cav1.4^−/−^ retina (Figure 5B). Since we previously reported that the secretion of RS1 depends on LTCCs in the chicken retina (Ko et al., 2008), these morphological results suggest that the reduction of calcium influx through LTCCs might dampen the expression or secretion of RS1 from photoreceptors. Alternatively, since Cav1.4 is necessary for ribbon synapse formation, the deletion of Cav1.4 may cause structural defects and decreased numbers of ribbon synapses, which might diminish the amount of RS1, a binding partner of Cav1.4.

### Deletion of Cav1.3 Also Decreased RS1 in Mouse Retinas

**Figure 6:**
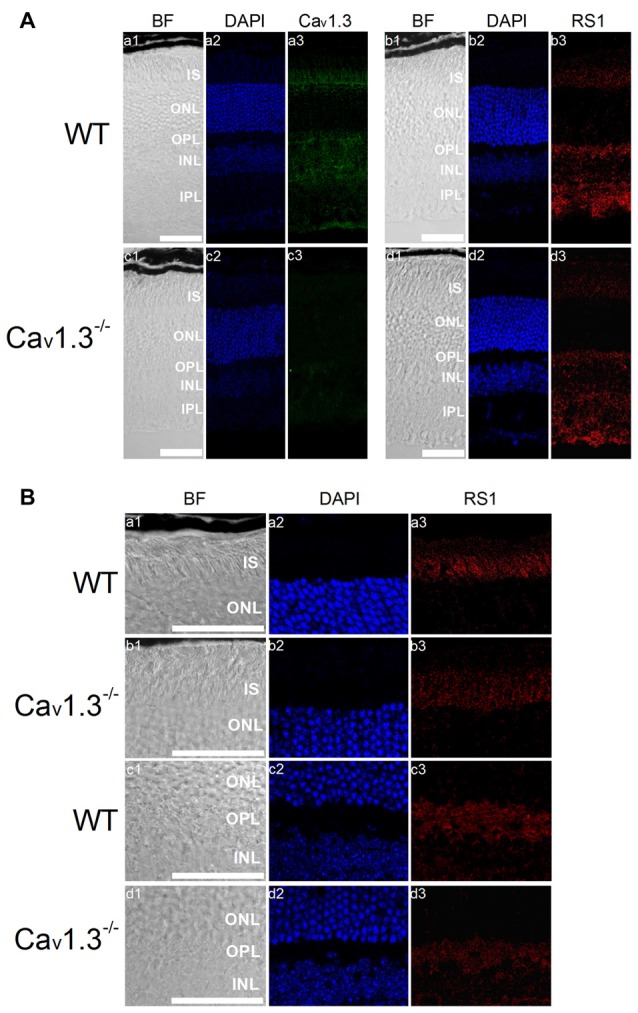
Deletion of Cav1.3 decreases RS1 distribution in the retina. **(A)** Images were taken at a lower magnification (20×) of WT (a1-a3, b1-b3) and Cav1.3^−/−^ (c1-c3, d1-d3). Retinal sections were immunostained for Cav1.3 (a1-a3, c1-c3) and RS1 (b1-b3, d1-d3). The scale bar represents 50 μm. **(B)** Images were taken at a higher magnification (40×) of WT (a1-a3, c1-c3) and Cav1.3^−/−^ (b1-b3, d1-d3). Retinal sections were immunostained for RS1. The scale bar represents 50 μm. DAPI stains the nuclei. BF, bright field; IS, photoreceptor inner segments; ONL, outer nuclear layer; OPL, outer plexiform layer; INL, inner nuclear layer; IPL, inner plexiform layer.

The retina of Cav1.3-null mutant (Cav1.3^−/−^) mice displays mild morphological changes in the OPL and slightly dampened light responses (Busquet et al., 2010), but these animals still have vision. However, it is not clear whether deletion of Cav1.3 might affect RS1 distribution in the retina, so we next examined the distribution of RS1 in Cav1.3^−/−^ retinas. Cav1.3 and RS1 were both present in all major layers of the WT retina (Figure 6A). Deletion of Cav1.3 (Cav1.3^−/−^) dampened the RS1 signal in the IS of photoreceptors and OPL (Figures 6A,B), suggesting that reduction of calcium influx due to a lack of Cav1.3-LTCCs decreased the expression and/or secretion of RS1 from photoreceptors and bipolar cells. An alternative explanation is that normal interaction between Cav1.3 and RS1 is necessary for the retention of both on the plasma membrane.

## Discussion

RS1, an extracellular adhesion protein with 224 amino acids, is essential for maintaining retinal cyto-architecture (Reid et al., 1999; Molday, 2007). Even though RS1 binds to other membrane proteins including ion channels and ATPase, the functional importance of these interactions are underestimated. We found that RS1 interacted with the first motif (I) from the N-terminals of Cav1.3 (Shi et al., 2009) and Cav1.4 (Figure 1). RS1 enhanced the channel conductance and voltage-dependent activation of Cav1.3- and Cav1.4-LTCCs without affecting the CDI properties, which further confirmed that RS1 did not interact with the C-terminal end of LTCCs. We previously showed that RS1 significantly enhances plasma membrane insertion and retention of LTCCs in chicken photoreceptors (Shi et al., 2009), which could also explain why co-transfection of RS1 and LTCCs in HEK cells had significantly larger LTCC currents compared to singular transfections of LTCCs (Figures 2, 3). In RS1^−/−^ mouse retinas, the expression of both Cav1.3 and Cav1.4 markedly decreased (Figure 4), while in Cav1.4^−/−^ or Cav1.3^−/−^ retinas, RS1 expression was also dampened (Figures 5, 6). Deletion of RS1 or Cav1.4 causes developmental deficits and degeneration of the retina, so the downregulation of Cav1.3 and Cav1.4 due to RS1 mutations or downregulation of RS1 due to the Cav1.4 null mutation would be expected. However, we did not expect to observe that there was a global decrease of RS1 expression in the Cav1.3^−/−^ retinas, since Cav1.3 null mutation does not cause retinal degeneration (Busquet et al., 2010). Interestingly, in retinal photoreceptors, after RS1 is secreted, it densely accumulates around the ISs (Vijayasarathy et al., 2007) where the Cav1.3-LTCCs are also expressed (Firth et al., 2001; Xu et al., 2002; Morgans et al., 2005; Ko et al., 2007). Hence, we postulate that the physical interaction between Cav1.3 and RS1 might contribute to the extracellular retention of RS1 on the plasma membrane, while RS1 clearly enhances the membrane retention of Cav1.3 (Ko et al., 2008; Shi et al., 2009).

While the role of Cav1.4 in retinal synaptic transmission is well-defined, and mutations in Ca_v_1.4 cause X-linked incomplete CSNB2 in humans (Bech-Hansen et al., 1998; Liu et al., 2013), the role of Cav1.3 in the retina is less known. In WT, we found that Cav1.3 was present from the photoreceptor ISs to the inner plexiform layer (IPL), which is similar to a previously published result (Busquet et al., 2010). The distribution of Cav1.3 in the IPL is consistent with previous findings that Cav1.3 is expressed in the lobular appendages of AII amacrine cells (Habermann et al., 2003) and is responsible for glycine release from these cells (Balakrishnan et al., 2015). Thus, it is possible that Cav1.3^−/−^ retinas might have impaired crossover inhibition from amacrine cells. However, the ERG recorded from Cav1.3^−/−^ mice (Busquet et al., 2010) only shows a mild decrease of the b-wave. Thus far, there is no report on the possible visual deficit in Cav1.3^−/−^ mice, which will require future investigations. Nonetheless, mice lacking Cav1.3 exhibit bradycardia and arrhythmia due to sinoatrial node dysfunction (Platzer et al., 2000; Namkung et al., 2001). Furthermore, Cav1.3^−/−^ mice are deaf (Platzer et al., 2000), since Cav1.3 is responsible for glutamate release from the inner hair cells in the cochlea (Platzer et al., 2000; Inagaki and Lee, 2013). Currently, there is no evidence that RS1 or RS1-like molecules are expressed in the cochlea. This might be due to a major structural difference, in which the retina is a multi-layered structure, but the organ of Corti is not. In the retina, RS1 is important in serving as an extracellular anchoring protein to stabilize the overall retinal architecture (Reid et al., 2003; Wu and Molday, 2003; Vijayasarathy et al., 2012; Ziccardi et al., 2012), since photoreceptor outer segments are constantly shed and renewed, and synaptic terminals undergo ultrastructural changes in response to ambient illumination and circadian control (Anderson et al., 1978; Baylor and Lamb, 1982; Burnside et al., 1982; Remé et al., 1986; Cahill and Besharse, 1993; Manglapus et al., 1998; Green and Besharse, 2004; Hull et al., 2006b; Ko et al., 2007; Tosini et al., 2008; Shi et al., 2009). In addition, there is a retinomotor movement in the photoreceptors that is also affected by the light and circadian control (Besharse et al., 1982; Burnside and Ackland, 1984; Burnside, 2001; Menger et al., 2005). Thus, the functional interaction between RS1 and LTCCs in the retina not only plays a role in modulating the gating properties of LTCCs, this interaction further enhances the membrane retention of each other (Ko et al., 2008; Shi et al., 2009) and maintains the structural stability of the retina.

Besides LTCCs, RS1 is known to interact with other molecules on the plasma membrane such as phosphatidylserine (Kotova et al., 2010) and the protein complex of sodium/potassium ATPase with the sterile alpha and TIR motif-containing protein 1 (Na/K-ATPase-SARM1 complex; (Molday et al., 2007). The interaction between RS1 and phosphatidylserine appears to be Ca^2+^ dependent (Kotova et al., 2010). A study using an artificial lipid bilayer and atomic force microscopy suggests that RS1 is partially embedded into the lipid bilayer (Kotova et al., 2010). We showed that RS1 interacts with the first 500 amino acids of the N-terminal of Cav1.3 (Shi et al., 2009) and Cav1.4 (Figure 1), which contains the first motif (I) of LTCCα1. If RS1 is partially embedded in the plasma membrane, it is likely that its interaction with LTCCα1 is not limited to the extracellular surface, but the exact configuration of the interaction between RS1 and LTCCs will require further investigation. RS1 is known to form homologous oligomers because of its discoidin domain (Wu and Molday, 2003; Bengert and Dandekar, 2005). Using Western blot analyses, RS1 dimers are often observed at ~26 kD due to the reducing agents often used in the procedure. Without reducing agents, the band for RS1 is above ~200 kD (Bush et al., 2016). Two recent studies applying high resolution cryo-electron microscopy (cryo-EM; Tolun et al., 2016) or single molecule EM (Bush et al., 2016) revealed that RS1 forms a double-octameric ring in the shape of a double-cogwheel. Intermolecular disulfide bonds are present in the inner ring to form the core octameric structure (Bush et al., 2016). However, whether it is the RS1 dimers or the whole cogwheel complex that interacts with other proteins including LTCCs remains to be investigated.

While XLRS and CSNB2 retinas have different morphological phenotypes, and XLRS patients have schisis while CSNB2 patients do not, their ERGs share several similarities, including more severely dampened cone responses and negative b-waves (Bradshaw et al., 2004; Mansergh et al., 2005; Sikkink et al., 2007). One potential explanation is that mutations in RS1 cause decreases in LTCCs thus leading to decreased synaptic transmission and lowered ERG b-wave (Shi et al., 2009; Bush et al., 2016). The lack of functional Cav1.4 in the retina of CSNB2 patients causes severely diminished synaptic transmission, so these patients have negative ERG b-waves. Here, we provide the first evidence that RS1 and Cav1.4 physically and functionally interact with each other. We postulate that in the case of XLRS, without RS1, there would be a decrease of plasma membrane insertion and retention of LTCCs, which causes a failure in establishing synaptic connections and leads to structural disorganization and retinal degeneration, since LTCCs are essential in the formation of synapses during development (Liu et al., 2013). We do not know why Cav.1.4^−/−^ mouse retinas have decreased RS1, since Cav1.4 deletion should only impact the development of photoreceptor synapses (Liu et al., 2013) without hampering RS1 synthesis or extracellular distribution around ISs of photoreceptors. One possibility is that the RS1 release from photoreceptors depends on the calcium influx from Cav1.4 in mammalian retinas, since we previously showed that the secretion of RS1 is dependent on LTCCs in avian retinas (Ko et al., 2008; Shi et al., 2009). Since the genes encoding RS1 and Cav1.4 are both located on the X chromosome in humans, we cannot rule out an alternative possibility that mutations in one gene might affect the expression of the other. This will require future investigations in human patients as well as appropriate animal models. Even though there is no scientific or clinical report available on human patients suffering from both XLRS and CSNB2 thus far, we suspect that there could be rare cases of concurrent mutations of RS1 and Cav1.4 (CACNA1F), where the two genes are indeed affecting the expressions of each other.

Taken together, we provide the first insight on the physical and functional interactions between RS1 and LTCCs (Cav1.3 and Cav1.4) in mammalian retinas. We demonstrated how RS1 enhanced the currents, channel conductance and voltage-dependent activation of LTCCs without changing the CDI property of LTCCs in HEK cells. Furthermore, in RS1^−/−^ retinas, the expressions of Cav1.3 and Cav1.4 were dampened, and in Cav1.4^−/−^ retinas, the presence of RS1 was also decreased. Our findings show the significance of the bi-directional interaction between RS1 and LTCCs.

## Author Contributions

LS and GY-PK conceived and designed the experiments. LS and MLK conducted the experiments. MLK performed the co-immunoprecipitation. LS conducted the HEK cell transfections, patch-clamp recordings and immunohistochemistry. LS and GY-PK analyzed the data. GY-PK provided experimental materials and animals. LS, MLK and GY-PK wrote the manuscript.

## Conflict of Interest Statement

The authors declare that the research was conducted in the absence of any commercial or financial relationships that could be construed as a potential conflict of interest.
